# Metabolic profiling of milk thistle different organs using UPLC-TQD-MS/MS coupled to multivariate analysis in relation to their selective antiviral potential

**DOI:** 10.1186/s12906-024-04411-7

**Published:** 2024-03-07

**Authors:** Alaa A. El-Banna, Reham S. Ibrahim

**Affiliations:** Department of Pharmacognosy, Faculty of Pharmacy, Alexandria University, Alexandria, 21521 Egypt

**Keywords:** Milk thistle, Selectivity index, Human coronavirus (HCoV-229E), Metabolomics, LC-MS/MS

## Abstract

**Introduction:**

*Silybum marianum* commonly known as milk thistle is one of the most imperative medicinal plants due to its remarkable pharmacological activities. Lately, the antiviral activities of *S. marianum* extract have been studied and it showed effectiveness against many viruses.

**Objective:**

Although most previous studies were concerned mainly with silymarin content of the fruit, the present study provides comprehensive comparative evaluation of *S. marianum* different organs’ chemical profiles using UPLC-MS/MS coupled to chemometrics to unravel potentially selective antiviral compounds against human coronavirus (HCoV-229E).

**Methodology:**

UPLC-ESI-TQD-MS/MS analysis was utilized to establish metabolic fingerprints for *S. marianum* organs namely fruits, roots, stems and seeds. Multivariate analysis, using OPLS-DA and HCA-heat map was applied to explore the main discriminatory phytoconstituents between organs. Selective virucidal activity of organs extracts against coronavirus (HCoV-229E) was evaluated for the first time using cytopathic effect (CPE) inhibition assay. Correlation coefficient analysis was implemented for detection of potential constituents having virucidal activity.

**Results:**

UPLC-MS/MS analysis resulted in 87 identified metabolites belonging to different classes. OPLS-DA revealed in-between class discrimination between milk thistle organs proving their significantly different metabolic profiles. The results of CPE assay showed that all tested organ samples exhibited dose dependent inhibitory activity in nanomolar range. Correlation analysis disclosed that caffeic acid-*O*-hexoside, gadoleic and linolenic acids were the most potentially selective antiviral phytoconstituents.

**Conclusion:**

This study valorizes the importance of different *S. marianum* organs as wealthy sources of selective and effective antiviral candidates. This approach can be extended to unravel potentially active constituents from complex plant matrices.

**Supplementary Information:**

The online version contains supplementary material available at 10.1186/s12906-024-04411-7.

## Introduction

*Silybum marianum* (L.) Gaertn is an annual or biennial plant belonging to family Asteraceae [1]. It has many common names, the most widely known one is milk thistle [2]. It is native to the Mediterranean districts of Northern Africa, Southern Europe, and Western Asia, but now it is cultivated throughout the whole world [3], either as vegetable, medicinal or as ornamental plant [4].

Owing to its various beneficial effects, *S. marianum* is among the most-selling botanical dietary supplements worldwide with an average sale of about US$ 8 billion/annum [5]. Recently, the milk thistle supplements market has globally expanded due to the COVID-19 outbreak, that led to increased need for immunomodulating supplements, in addition to the growing demand of effective anti-inflammatory, anti-aging and skin care natural products. In foods, its leaves and flowers are consumed as a vegetable for salads and a substitute for spinach. Milk thistle seeds can be used in raw form or made into tea. They can be also roasted for use as a coffee substitute [6].

*S. marianum* has been utilized as a medicinal plant of long ago, mainly for mitigation of liver, kidney, spleen and gall bladder diseases [7, 8]. Nowadays, its extract is sold in the market under many brand names due to its many reported and astonishing pharmacological activities [9]. It has been proved to possess antioxidant [10], hepatoprotective [11], anticancer [12], anti-inflammatory [13], anti-diabetic [14], anti-amnesia [15], antiplatelet [16] and cardioprotective [17] activities. It has also been utilized for alleviation of obsessive-compulsive disorder [18], depression [19], menstrual disorders and varicose veins [20].

Lately, the antiviral activities of *S. marianum* extract have been studied and it showed effectiveness against many viruses such as the flaviviruses (hepatitis C virus and dengue virus) [21, 22], human immunodeficiency virus [23], togaviruses (Chikungunya virus and Mayaro virus) [24, 25], hepatitis B virus [26] and influenza virus [27]. The remarkable antiviral efficacy of *S. marianum* extract is attributed to its multi-target activity against host cell. As it showed ability to modulate cell innate immunity [28, 29], inflammation [30], oxidative stress [31] and autophagy [32], which are cellular processes impaired by the viral invasion. In addition to the modulation of the cell environment, *S. marianum* extract also showed ability to exert direct potent antiviral actions against viral proteins [33]. These findings encouraged the researchers to assess the effectiveness of *S. marianum* against severe acute respiratory syndrome coronavirus 2 (SARS-CoV-2), the causative agent of COVID-19 pandemic. It was computationally found to act as inhibitor of signal transducer and activator of transcription 3 (STAT3), the main modulator of inflammatory and immune response. In addition, it was predicted to inhibit RNA-dependent RNA polymerase (RdRp), the main protein responsible for SARS-CoV-2 replication and transcription [34].

The first human coronavirus (HCoV) strain was found out in 1965. Afterwards, additional 30 strains were recognized, from which HCoV-229E was the prototypic strain that HCoV research focused on until 2002–2003, where severe acute respiratory syndrome coronavirus (SARS-CoV) was flared up. Thereafter, the Middle East respiratory syndrome coronavirus (MERS-CoV) and the 2019 novel coronavirus (SARS-CoV-2) have broken out [35, 36]. Differently from SARS-CoV, MERS-CoV and SARS-CoV-2 that bring about severe respiratory disease, HCoV-229E usually leads to mild to moderate upper-respiratory tract ailment, contributing to about 15–30% of human common cold cases [35]. HCoV-229E is an enveloped, single-stranded RNA virus. It is a member of *Alphacoronavirus* genus and *Duvinacovirus* subgenus [37].

*S. marianum* is rich in diverse secondary metabolites, including silymarin (which is a mixture of flavonolignans), phenolics, fatty acids and other chemical constituents [38]. The majority of previous studies focus only on the phytochemical and biological investigation of the flavonolignans constituents of *S. marianum* seeds and fruits [39–48], and up to authors’ knowledge there are not previous work on studying the whole metabolome and antiviral activity of all different parts of milk thistle. Therefore, the study in hand aims to investigate the whole chemical profile of different *S. marianum* organs including fruits, leaves, stems and roots using HPLC-MS/MS and chemometric analysis for the first time and to couple these data with the antiviral activity of these organs aiming at valorizing the unused milk thistle parts. The orthogonal projections to latent structures discriminant analysis (OPLS-DA) was performed to examine the class discrimination between the tested extracts and reveal the chemical markers accountable for such discrimination. Afterwards, the antiviral potentials of the tested extracts against HCoV-229E were determined on African green monkey kidney (Vero E6) cells using cytopathic effect (CPE) inhibition assay. Thereafter, different chemometric models were constructed to identify the biological markers responsible for the bioactive segregation of the studied extracts to exploit them as potential sources of valuable antiviral agents.

## Experimental

### Collection of the plant material

Five separate samples of the plant material were collected during the flowering-fruiting stage from farms belonging to the Faculty of Agriculture, Alexandria University, Egypt, in July 2022. The plant identity was confirmed via comparison with herbal sample present in the herbarium of the Faculty of Science, Alexandria University, Egypt. A voucher specimen (SM2022) was held at the Department of Pharmacognosy-Faculty of Pharmacy-Alexandria University. The collected plant materials were allowed to dry at room temperature prior to phytochemical analysis.

### Preparation of samples

Every plant sample was split up into four organs: fruits, leaves, roots, and stems. Every organ sample (100 g) was extracted individually by ultrasonication in 200 mL of 70% ethanol using an ultrasonic bath 28 kHz/1100 W for 30 min at 45 ºC twice. The filtrates of each organ were collected and evaporated to dryness using a rotary evaporator, under reduced pressure, at 45ºC to get a total of 20 samples.

###  Profiling the metabolome of *S. marianum* different organs extracts using UPLC-MS/MS

#### Samples preparation for UPLC-MS analysis

Methanolic solutions with concentrations of 1 mg ml^−1^ were prepared for each sample. These solutions were subjected to filtration via membrane filters (0.2 μm) and degassing by sonication before being analyzed via LC-MS. To ensure reproducibility, the above process was performed three times for every sample.

#### UPLC-ESI- TQD -MS analysis

##### Chromatographic parameters and conditions

The UPLC system consists of a Waters Acquity QSM pump, an LC-2040 (Waters) autosampler, degasser and Waters Acquity CM detector. 10 µL of each of the previously prepared samples (full loop injection volume) were separately injected into the chromatographic column three times. Chromatographic separation was conducted using a Waters Acquity UPLC BEH C18 column (50 mm × 2.1 mm ID × 1.7 μm particle size) operating at a flow rate of 0.2 mL/min and thermostating at 30 °C.

The analyses were performed using a binary mobile phase consisting of ultrapure water + 0.1% (v/v) formic acid (Phase A) and methanol + 0.1% (v/v) formic acid (Phase B). The mobile phase was prepared by filtration using 0.2 μm membrane disc filter and degassed by sonication before injection. It was pumped at 0.2 mL/min into the UPLC system. The mobile phase gradient elution was programmed as follows: 0.0–2.0 min, 10% B; 2.0–5.0 min, 30% B; 5.0–15.0 min, 70% B; 22.0 min, 90% B; 22.0–25.0 min, 90% B; 26.0 min, 100% B; 26.0–29.0 min, 100% B; 30.0 min, 10% B; followed by 4 min of re-equilibration.

##### ESI-MS parameters and conditions

For LC/MS analysis, a triple quadrupole mass spectrometer was coupled to the UPLC instrument via an ESI interface. Ultra-high purity helium (He) was used as the collision gas and high purity nitrogen (N2) as the nebulizing gas. The mass spectrometer was monitored in negative ionization mode over 50–1200 m/z mass range. The optimized detection parameters were as follows: temperature 150 °C, cone voltage 30 V, capillary voltage 3 kV, desolvation temperature 440 °C, cone gas flow 50 L/h, and desolvation gas flow 900 L/h. A source fragmentation voltage of 25 V was applied. The mass spectrometer was operated in negative ion mode in order to identify the molecular ions [M-H]^−^ followed by MS/MS product ion experiments to study the fragmentation pattern of the constituents. The analysis process run time lasted for 40 min. Regarding automatic MS/MS fragmentation process of the precursor ions that have been filtered by the first quadrupole (Q1), the mass fragmentation was performed through collision-induced dissociation (CID) energy utilizing Ultra-high purity helium in the second quadrupole (Q2). Eventually, the third quadrupole mass analyzer (Q3) filtered the daughter ions produced from CID that consequently related to the molecular structure of the precursor ions. The collision energy for CID in tandem mass spectrometry analysis, was optimized for each compound, in order to acquire mass spectra with various fragmentation degrees from the precursor ion thus attaining as much structural information as possible. A data-dependent program was utilized for tandem mass spectrometry data acquisition. In this program, molecular ions detected in the negative ion mode were selected for MS2 analysis and the two most abundant fragment ions in the MS2 spectra were then selected for further MS3 fragmentation.

#### Annotation of UPLC-MS/MS compounds

The raw UPLC–MS data were pre-processed using Mzmine® version 2.8 software that has been utilized for importing data, chromatogram building, peak deconvolution, alignment and annotation. Tentative assignment of metabolites was established via comparing their retention times relative to standards (which were caffeic acid, malic acid, quercetin, coumarin, *p*-coumaryl alcohol, lanosterol, and linoleic acid that were used as standards to their respective chemical classes), interpreting tandem mass spectra (quasi-molecular ions as well as diagnostic MS/MS fragmentation profiles) combined with our in-house comprehensive database that was set up covering all compounds previously reported in the literature including Dictionary of Natural Products (https://dnp.chemnetbase.com/), PubChem and MassBank (https://massbank.eu/MassBank/) to provide high confidence level of annotation [49, 50].

#### Semi-quantitation of identified compounds using UPLC-MS/MS

The annotated compounds were semi-quantified in accordance with their chemical class by the use of standard compound solutions. Caffeic acid, malic acid, quercetin, coumarin, *p*-coumaryl alcohol, lanosterol, and linoleic acid were used as standards for their chemical classes, and they were procured from Sigma–Aldrich (St. Louis, Mo., USA). Stock methanolic solutions, each with concentration of 1 mg ml^−1^, were prepared for every standard compound. These stock solutions were then diluted to generate working concentrations extending from 0.0125 to 0.625 mg mL^−1^ using HPLC-grade methanol (Table 1). Each standard solution concentration was analyzed three times under the previously described conditions in UPLC-ESI- TQD -MS analysis section. The standards were analyzed in the same order shown in Table 1: caffeic acid, then malic acid, quercetin, coumarin, *p-*coumaryl alcohol, lanosterol, then linoleic acid. The calibration curves were constructed by plotting standards peak areas versus their concentrations. For each calibration curve, the equation is y = ax + b, where y is the peak area, x is the concentration of the standard (mg mL^−1^), a is the intercept, b is the slope and r is the correlation coefficient.

**Table 1:** Linearity and sensitivity parameters for caffeic acid, malic acid, quercetin, p-coumaryl alcohol, coumarin, lanosterol and linoleic acid used as *S. marianum* standards

	Standard compounds	Linearity range (mg mL^−1^)	Slope (b)	Intercept (a)	r	LOD (mg mL^−1^)	LOQ (mg mL^−1^)
1	Caffeic acid	0.0125–0.25	32*10^5^	35*10^7^	0.996	0.005	0.0125
2	Malic acid	0.025–0.536	2.87*10^8^	-8.35*10^9^	0.993	0.010	0.025
3	Quercetin	0.02–0.525	20.51*10^6^	-1.07*10^8^	0.995	0.012	0.02
4	Coumarin	0.055–0.625	5170	8.15*10^8^	0.994	0.003	0.0325
5	p-Coumaryl alcohol	0.0325–0.625	41.71*10^6^	-10.39*10^8^	0.997	0.015	0.055
6	Lanosterol	0.0125-0.25	1317.3	84.58*10^6^	0.993	0.005	0.0125
7	Linoleic acid	0.0135–0.255	30.71*10^2^	14.99*10^6^	0.995	0.003	0.0135

### Multivariate statistical analysis

Semiquantitative analysis and biological activity testing were statistically analyzed via ANOVA (one-way analysis of the variance) hiring SPSS 26.0 program (SPSS Inc., Chicago, IL. USA) and Metaboanalyst 4.0 (http://www.metaboanalyst.ca/) which is a web-based tool for processing metabolomics data to construct hierarchical cluster analysis (HCA) heat maps.

In addition, SIMCA v 14 software (Umetrics, Sweden) was applied for the construction of Orthogonal Projections to Latent Structures-Discriminant Analysis model (OPLS-DA) followed by Orthogonal Projections to Latent Structures (OPLS) model that enabled the discrimination of different milk thistle organs extracts based on their chemical profile in addition to antiviral activity. OPLS-DA model enabled the identification of the phytoconstituents that generated such discrimination. Meanwhile, careful examination of the OPLS correlation coefficient plots enabled us to identify the metabolites strongly correlated to the investigated biological activity. Permutations plots were created to validate that the created models were not modelling the noise or over-fitted.

### Selective virucidal activity of *S. marianum* different organs extracts against human coronavirus (HCoV-229E) using cytopathic effect (CPE) inhibition assay

The crystal violet method was used to evaluate antiviral and cytotoxic activities according to Schmidtke et al. (2001) [51]. In brief, Vero E6 cells (Nawah-Scientific, Egypt) were seeded into a 96-well plate at a density of 2 × 10^4^ cells/well one day before infection. Vero E6 cells were cultured in DMEM with 10% fetal bovine serum (FBS) and 0.1% antibiotic/antimycotic solution provided by Gibco BRL (Grand Island, NY, USA). After removing the culture medium the next day, the cells were washed with phosphate-buffered saline. Determination of coronavirus 229E (Nawah-Scientific, Egypt) infectivity was performed using the crystal violet method to monitor CPE and calculate the percentage of cell viability. 0.1 mL of diluted viral suspension of 229E virus with CCID 50 (50% cell culture infective dose of virus stock) was added to mammalian cells to attain the desired CPE after infection. Regarding samples’ treatments, 0.01 mL of desired extract-containing medium was added to the cells. Each test sample’s antiviral activity was estimated by a two-fold diluted concentration range of 0.1–100 µg/mL. The virus controls (virus-infected, non-drug-treated cells) and cell controls (non-infected, non-drug treated cells) were used. For 3 days, culture plates were incubated at 37^o^C in 5% carbon dioxide. The development of CPE was monitored by light microscopy. Following a PBS wash, fixation then staining of the cell monolayers was done using a 0.03% crystal violet solution in 2% EtOH and 3% formalin. Following washing and drying the optical densities (OD) of individual wells were quantified spectrophotometrically at 540/630 nm. The percentage of antiviral activity of the tested extracts was calculated by Pauwels et al. (1988) method [52], adopting the following equation:

$$\%\;\mathrm{Antiviral}\;\mathrm{activity}=\left[(\mathrm{mean}\;\mathrm{OD}\;\mathrm{of}\;\mathrm{cell}\;\mathrm{control}\:-\:\mathrm{mean}\;\mathrm{OD}\;\mathrm{of}\;\mathrm{virus}\;\mathrm{control})/(\mathrm{OD}\;\mathrm{of}\;\mathrm{test}\:-\:\mathrm{mean}\;\mathrm{OD}\;\mathrm{of}\;\mathrm{virus}\;\mathrm{control})\right]\times100$$

Prior to conducting this assay, we assessed the cytotoxicity on normal cells, cells were seeded at a density of 2 × 10^4^ cells/well in 96-well plate. The next day, the serially diluted extracts- containing culture media were added to the cells then incubated for 48 h then removed and the cells were washed with PBS. The following steps were performed as previously illustrated in the antiviral activity assay. GraphPad PRISM V 8 (San Diego, USA) software was used for determination of 50% cytotoxic concentrations (CC50) and 50% inhibitory concentrations (IC50).

## Results and discussion

### Characterization of metabolites in *S. marianum* different organs

**Fig. 1:**
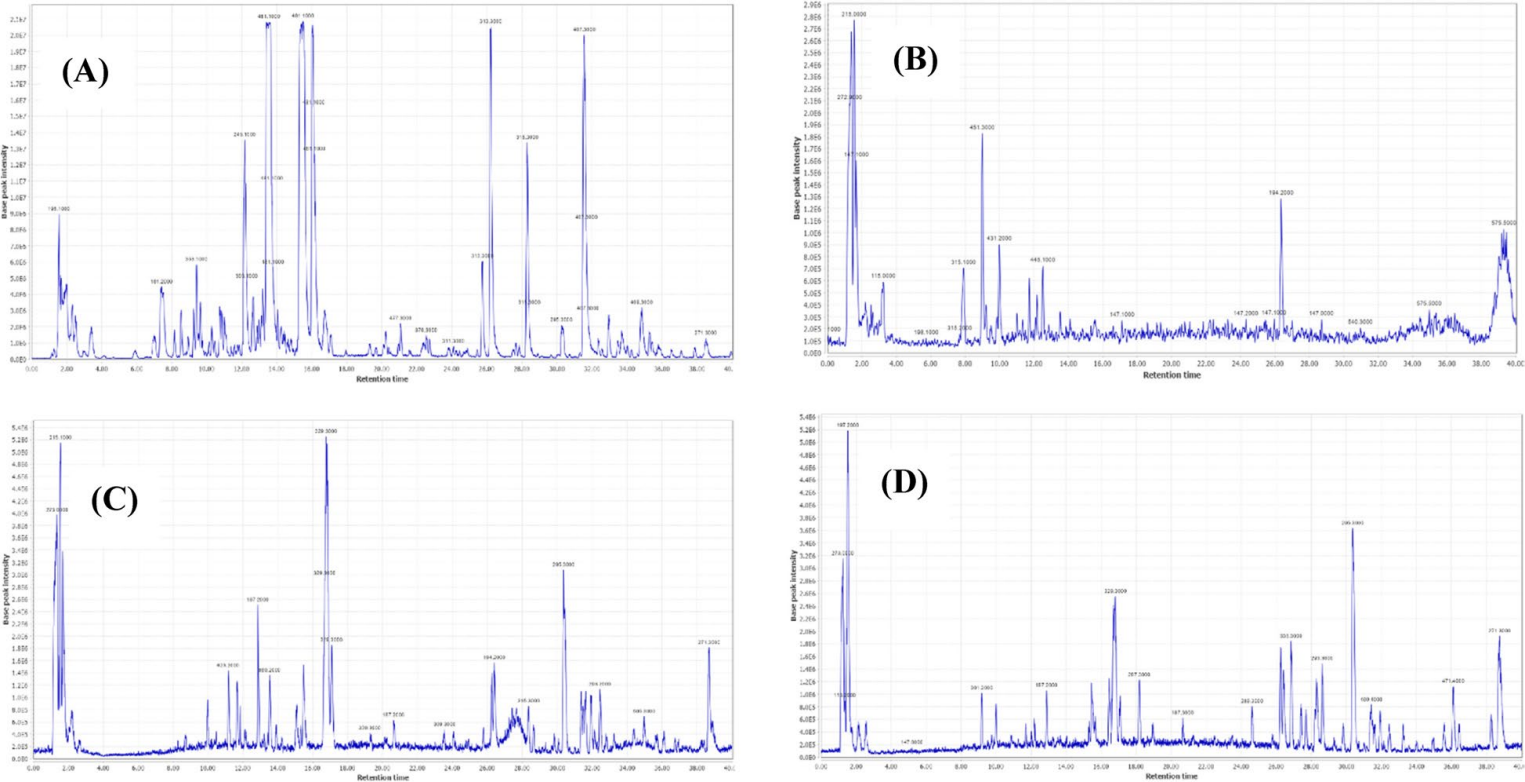
UPLC-ESI- TQD-MS base peak chromatograms of *S. marianum *extracts in negative ionization mode. Fruit (**A**), leaves (**B**), root (**C**) and stem (**D**) chromatograms

Metabolite profiling of *S. marianum* tested extracts was accomplished using UPLC-MS-MS. Figure 1 represents the base peak chromatograms of the studied extracts from which 87 metabolites were tentatively annotated by their retention times comparison to references and examining their MS data (Table 2). The details of characterization and fragmentation patterns of the identified metabolites are illustrated below.

**Table 2:** Metabolites identified in different organs of *S. marianum* extracts using UPLC-MS in negative ionization mode

No.	Rt (min.)	[M-H]^−^	Element composition	MS^n^ ions m/z (-)	Identified compounds	Chemical class	Structure	Reference
1	1.36	331.25	C13H16O10	169.11, 125.11	Gallic acid hexoside	Phenolic acid glycoside		Li et al. 2003 [53]
2	1.57	327.26	C14H16O9	312.23, 192.13, 164.12	Bergenin	Phenolic acid glycoside		Li et al. 2013 [54]
3	1.69	341.29	C15H18O9	179.15, 135.15	Caffeic acid-*O*-hexoside	Phenolic acid glycoside		Singh et al. 2016 [55]
4	1.81	191.16	C7H12O6	147.16	Quinic acid	Phenolic acid		Haşimi et al. 2017 [56]
5	2.01	153.11	C7H6O4	109.11	Protocatechuic acid	Phenolic acid		Haşimi et al. 2017 [56]
6	2.32	167.14	C8H8O4	152.11, 123.14	Vanillic acid	Phenolic acid		Haşimi et al. 2017 [56]
7	2.48	197.17	C9H10O5	182.14, 153.17	Syringic acid	Phenolic acid		Haşimi et al. 2017 [56]
8	2.66	179.15	C9H8O4	135.15	Caffeic acid^a^	Phenolic acid		Haşimi et al. 2017 [56]
9	7.91	163.15	C9H8O3	119.15	*p-*Coumaric acid	Phenolic acid		Haşimi et al. 2017 [56]
10	8.70	193.18	C10H10O4	178.15, 149.18	Ferulic acid	Phenolic acid		Haşimi et al. 2017 [56]
11	9.98	147.15	C9H8O2	103.15	Cinnamic acid	Unsaturated carboxylic acid		Haşimi et al. 2017 [56]
12	10.14	353.3	C16H18O9	179.15, 191.16	Chlorogenic acid	Phenolic acid		Haşimi et al. 2017 [56]
13	10.85	275.28	C15H16O5	201.25	Ursinoic acid	Terpenoid		Hines et al. 2017 [57]
14	11.01	133.08	C4H6O5	115.06 71.06	Malic acid^a^	Dicarboxylic acid		Al Kadhi et al. 2017 [58]
15	11.12	115.06	C4H4O4	71.06	Fumaric acid	Dicarboxylic acid		Al Kadhi et al. 2017 [58]
16	11.74	607.49	C27H28O16	313, 295, 285.23, 151.1, 133.12	Kaempferol 3,7-dihexoside	Flavonoid		Said et al. 2017 [59]
17	12.18	609.51	C27H30O16	301.23, 272.21, 255.2, 151.1	Rutin	Flavonoid		Kumar et al. 2017 [60]
18	12.22	593.5113	C27H30O15	269.23, 151.1, 117.13	Apigenin 7-dihexoside	Flavonoid		Liu et al. 2010 [61]
19	12.31	623.54	C28H32O16	315.26, 300.22, 271.2, 151.1	Isorhamnetin-3-*O*-dihexoside	Flavonoid		Krasteva and Nikolov, 2008 [62]
20	12.39	579.53	C27H32O14	271.25, 151.1, 119.14, 107.09	Naringin	Flavonoid		Santoro et al. 2020 [63]
21	12.45	325.29	C15H18O8	163.15, 119.14	Coumaroyl hexoside	Phenolic acid glycoside		Li et al. 2003 [53]
22	12.51	491.38	C22H20O13	315.26, 300.22, 271.2, 151.1	Isorhamnetin-3-*O*-hexuronide	Flavonoid		Krasteva and Nikolov, 2008 [62]
23	12.71	447.37	C21H20O11	285.23, 151.1, 133.12	Luteolin-7-*O*-hexoside	Flavonoid		Santoro et al. 2020 [63]
24	12.80	477.4	C22H22O12	325, 315.26, 300.22, 271.2, 151.1	Isorhamnetin-3-*O*-hexoside	Flavonoid		Krasteva and Nikolov, 2008 [62]
25	12.89	431.37	C21H20O10	269.23, 151.1, 117.13	Apigenin-7-*O*-hexoside	Flavonoid		Liu et al. 2010 [61]
26	13.20	432.38	C21H21O10+	286.24, 151.1, 135.1	Cyanidin-3-*O*-deoxyhexoside	Flavonoid		Santoro et al. 2020 [63]
27	13.47	433.39	C21H22O10	271.25, 151.1, 119.14, 107.09	Naringenin 7-*O*- hexoside	Flavonoid		Santoro et al. 2020 [63]
28	13.53	457.36	C22H18O11	125.1, 169.11	(-)-Epigallocatechin gallate	Flavonoid gallic acid ester		Spáčil et al. 2010 [64]
29	13.91	461.40	C22H22O11	285.23, 151.1, 133.12	Rhamnocitrin-*O*-hexoside	Flavonoid		Said et al. 2017 [59]
30	14.21	415.37	C21H20O9	253.23, 135.1, 117.13	Daidzein-7-*O*-hexoside	Flavonoid		Santoro et al. 2020 [63]
31	14.44	441.37	C22H18O10	125.1, 109.1, 169.11	Catechin gallate	Flavonoid gallic acid ester		Spáčil et al. 2010 [64]
32	14.95	445.4	C22H22O10	283.26, 268.23, 224.23	Trifolirhizin	pterocarpanoid glycoside		Ye et al. 2012 [65]
33	15.10	473.41	C23H22O11	269.23, 151.1, 117.13	Apigenin 7- hexuronide, Et ester	Flavonoid		Liu et al. 2010 [61]
34	15.33	429.4	C22H22O9	267.26, 132.16, 135.1	Ononin	Flavonoid		Santoro et al. 2020 [63]
35	15.48	519.52	C26H32O11	357.38, 339.36, 221.23, 191.2	Dehydrodiconiferyl alcohol-hexoside	Neolignan		Letourneau and Volmer, 2021 [66]
36	15.54	161.13	C9H6O3	133.12	4-Hydroxycoumarin	Coumarin derivative		Ren et al. 2016 [67]
37	15.62	145.14	C9H6O2	117.13	Coumarin^a^	O-hydroxycinnamic acid (Aromatic compound)		Ren et al. 2016 [67]
38	15.80	149.17	C9H10O2	131.15	*p*-Coumaryl alcohol^a^	Monolignol		Liu et al. 2010 [61]
39	15.89	175.16	C10H8O3	147.15	4-Methylumbelliferone	Coumarin derivative		Ren et al. 2016 [67]
40	15.99	181.21	C10H14O3	163.19	2-Hydroxymethyl-5-(2-hydroxypropan-2-yl) phenol	Aromatic compound		Dictionary of Natural Products 30.2 Chemical Search
41	16.09	181.21	C10H14O3	163.19	*p-*Mentha-1,3,5-triene-2,7,8-triol	Aromatic compound		Dictionary of Natural Products 30.2 Chemical Search
42	16.13	217.2	C12H10O4	175.16	4-Methylumbelliferyl acetate	Coumarin derivative		Leonart et al. 2017 [68]
43	16.31	301.23	C15H10O7	272.21, 255.2, 151.1	Quercetin^a^	Flavonoid		Kumar et al. 2017 [60]
44	16.43	303.24	C15H12O7	285.22, 275.23, 151.1, 125, 175	Taxifolin (Dihydroquercetin)	Flavonoid		Chen et al. 2016 [69]
45	16.56	305.26	C15H14O7	125.1	Epigallocatechin	Flavonoid		Spáčil et al. 2010 [64]
46	16.61	285.23	C15H10O6	151.1, 133.12	Kaempferol	Flavonoid		Said et al. 2017 [59]
47	16.70	287.24	C15H12O6	151.1, 135.14	Dihydrokaempferol (Aromadendrin)	Flavonoid		Said et al. 2017 [59]
48	16.77	315.26	C16H12O7	300.22, 271.2, 151.1	Isorhamnetin	Flavonoid		Krasteva and Nikolov, 2008 [62]
49	16.89	269.23	C15H10O5	151.1, 117.13	Apigenin	Flavonoid		Liu et al. 2010 [61]
50	17.10	269.23	C15H10O5	241.22, 225.23	Genistein	Flavonoid		Ye et al. 2012 [65]
51	18.38	271.25	C15H12O5	151.1, 119.14, 107.09	Naringenin	Flavonoid		Santoro et al. 2020 [63]
52	19.27	273.26	C15H14O5	151.1, 121.16	Phloretin	Chalcone derivative		Bystrom et al. 2008 [70]
53	20.13	329.28	C17H14O7	314.25, 299.22, 271.21	Tricin	Flavonoid		Kang et al. 2016 [71]
54	20.63	283.26	C16H12O5	269.23, 151.1, 117.13	Genkwanin (7-methoxyapigenin)	Flavonoid		Liu et al. 2010 [61]
55	23.55	239.25	C15H12O3	130.14, 102.13	2’,4’-Dihydroxychalcone	Chalcone derivative		Zhang et al. 2014 [72]
56	23.87	207.25	C15H12O	130.14, 102.13	Chalcone	α,β-unsaturated ketone		Motiur Rahman et al. 2013 [73]
57	24.93	497.43	C25H22O11	463, 453, 179, 125, 480, 470, 375, 355	Silyamandin	Flavonolignan		Csupor et al. 2016 [74]
58	24.99	479.41	C25H20O10	151, 451.1, 255.03, 466.09	2,3-Dehydrosilybin	Flavonolignan		Csupor et al. 2016 [74]
59	25.05	481.43	C25H22O10	463, 453, 179, 125, 433, 423, 355, 337, 325	Silychristin	Flavonolignan		Csupor et al. 2016 [74]
60	25.23	481.43	C25H22O10	463, 453, 179, 125, 409, 151, 301	Silydianin	Flavonolignan		Csupor et al. 2016 [74]
61	25.47	481.43	C25H22O10	463, 453, 179, 125, 435, 301, 283, 273, 257, 423	Silybin A	Flavonolignan		Csupor et al. 2016 [74]
62	25.69	481.43	C25H22O10	463, 453, 179, 125, 435, 301, 283, 273, 257, 423	Silybin B	Flavonolignan		Csupor et al. 2016 [74]
63	25.81	481.43	C25H22O10	463, 453, 179, 125, 435, 301, 283, 273, 257	Isosilybin A	Flavonolignan		Csupor et al. 2016 [74]
64	25.99	481.43	C25H22O10	463, 453, 179, 125, 435, 301, 283, 273, 257	Isosilybin B	Flavonolignan		Csupor et al. 2016 [74]
65	26.05	323.36	C20H20O4	201.24	Glabridin	Flavonoid		Aoki et al. 2005 [75]
66	26.15	465.43	C25H22O9	151	Silandrin	Flavonolignan		Csupor et al. 2016 [74]
67	26.28	407.48	C25H28O5	119.14, 287.33	6,8-Diprenylnaringenin	Prenyl flavonoid		Nikolic and van Breemen, 2012 [76]
68	26.35	633.88	C37H62O8	471.74, 357.55, 339.53	24-Methylenelanost-8-ene-3,25,28-triol, 3-*O*-hexoside	Lanostane triterpenoid		Wu et al. 2011 [77]
69	26.41	483.66	C30H44O5	465.64, 421.64	Silymin A	Ursane triterpenoid		Ahmed et al. 2007 [78]
70	26.46	469.72	C31H50O3	371.53, 353.51	3,25-Dihydroxy-24-methylenelanost-8-en-7-one	Lanostane triterpenoid		Wu et al. 2011 [77]
71	26.50	179.19	C13H8O	151.18, 137.15	12-Tridecene-4,6,8,10-tetraynal	Unsaturated fatty aldehyde		Suzuki et al. 1980 [79]
72	26.58	179.19	C13H8O	164.16, 149.13	1,3-Tridecadiene-5,7,9,11-tetrayne, 1,2-epoxide	Unsaturated fatty epoxide		Suzuki et al. 1980 [79]
73	26.63	291.41	C18H28O3	273.39, 247.4	12-oxo-phytodienoic acid	Unsaturated fatty acid		Brenna, 2013 [80]
74	26.70	277.42	C18H30O2	259.4, 233.41	Linolenic acid	Unsaturated fatty acid		Brenna, 2013 [80]
75	26.74	279.44	C18H32O2	261.42, 235.43	Linoleic acid^a^	Unsaturated fatty acid		Brenna, 2013 [80]
76	26.83	281.45	C18H34O2	263.43, 237.44	Oleic acid	Unsaturated fatty acid		Brenna, 2013 [80]
77	26.88	241.35	C17H22O	223.33, 226.32	2,9,16-Heptadecatriene-4,6-diyn-8-ol	Unsaturated aliphatic alcohol		El Sayed et al. 2020 [81]
78	27.03	309.51	C20H38O2	291.49, 265.5	Gadoleic acid	Unsaturated fatty acid		Brenna, 2013 [80]
79	27.14	293.46	C19H34O2	262.43, 221.4, 81.14	Linoleic acid methyl ester	Unsaturated fatty acid methyl ester		Fagerquist et al. 1999 [82]; Härtig, 2008 [83]
80	27.18	295.48	C19H36O2	264.45, 221.4, 55.1	16-octadecenoic acid methyl ester	Unsaturated fatty acid methyl ester		Fagerquist et al. 1999 [82]; Härtig, 2008 [83]
81	27.24	227.36	C14H28O2	209.34, 183.35	Myristic acid	Saturated fatty acid		Brenna, 2013 [80]
82	27.32	255.42	C16H32O2	237.4, 211.41	Palmitic acid	Saturated fatty acid		Brenna, 2013 [80]
83	27.41	283.47	C18H36O2	265.45, 239.46	Stearic acid	Saturated fatty acid		Brenna, 2013 [80]
84	27.51	311.52	C20H40O2	293.5, 267.51	Arachidic acid	Saturated fatty acid		Brenna, 2013 [80]
85	27.66	339.58	C22H44O2	321.56, 295.57	Behenic acid	Saturated fatty acid		Brenna, 2013 [80]
86	27.88	295.52	C20H40O	277.5, 71.14	Phytol	Acyclic diterpene alcohol		El Sayed et al. 2020 [81]
87	28.30	409.63	C28H42O2	394.6, 379.57, 151.19	(R)-gamma-Tocotrienol	Fat soluble vitamin		Zhao et al. 2010 [84]

^a^The standards used for retention times comparison and semi-quantitation of the annotated compounds were caffeic acid, malic acid, quercetin, coumarin, *p-*coumaryl alcohol, lanosterol, and linoleic acid

#### Phenolics

The mass spectra of compounds **1**, **3** and **21** exhibited molecular ion peaks at m/z 331.25, 341.29 and 325.29, respectively. They were characterized as galloyl hexoside, caffeic acid-*O*-hexoside and coumaroyl hexoside respectively, due to the loss of hexoside (-162.14 Da) and CO_2_ (-44.01 Da) moieties and the presence of characteristic fragments at m/z 169.11 [M-H-hexoside]^-^ and m/z 125.11 [M-H-hexoside-CO_2_]^-^ for galloyl hexoside; at m/z 179.15 [M-H-hexoside]^-^ and m/z 135.15 [M-H-hexoside-CO_2_]^-^ for caffeic acid-*O*-hexoside; and at 163.15 [M-H-hexoside]^-^ and m/z 119.14 [M-H-hexoside-CO_2_]^-^ for coumaroyl hexoside [53, 55].

Whereas compound **2** having [M-H]^-^ at m/z 327.26 was annotated as bergenin. This annotation was suggested by the daughter peaks in the MS2 spectrum at m/z 312.23 representing the loss of methyl group, and the characteristic Retro Diels-Alder (RDA) fragment at m/z 192.13, which then lost carbonyl group to yield another fragment ion at m/z 164.12 [54].

Furthermore, the MS spectra of compounds **5**, **6**, **7**, **8**, **9**, **10** and **11** displayed [M–H]^-^ ions at m/z 153.11, 167.14, 197.17, 179.15, 163.15, 193.18 and 147.15, respectively, together with their decarboxylated fragments. For compounds **6**, **7** and **10** another fragment generated by methyl group (15.03 Da) loss was noticed in their MS2 spectra at m/z 152.11, 182.14, and 178.15, respectively. Therefore, these compounds were annotated as, protocatechuic acid (compound **5**), vanillic acid (compound **6**), syringic acid (compound **7**), caffeic acid (compound **8**), coumaric acid (compound **9**), ferulic acid (compound **10**) and cinnamic acid (compound **11**), respectively [56].

Moreover, the MS spectrum of compound **12** revealed [M–H]^−^ ion at m/z 353.3, in addition to characteristic daughter fragments at m/z 179.15 and 191.16 representing caffeic and quinic acids, respectively. Therefore, compound **12** was recognized as monocaffeoylquinic acid (chlorogenic acid) [56]. Also, quinic acid was present as free acid as shown in compound **4** spectrum which had a base peak [M-H]^−^ ion at m/z 191.16.

Compound **13** displaying a parent ion peak at m/z 275.28 was proposed to be ursinoic acid. This annotation was suggested by its MS/MS fragment ion at m/z 201.25 [M-H-44-30.03]^−^ owing to CO_2_ and methoxy group elimination [57].

#### Dicarboxylic acids

Two dicarboxylic acids were recognized. Malic acid (compound **14**) was proposed for the parent ion at m/z 133.08 which was then fragmented to yield peaks at m/z 115.06 and 71.06 due to successive loss of H_2_O and CO_2_. Whereas fumaric acid (compound **15**) was proposed for the parent ion at m/z 115.06 which decarboxylated to give fragment ion at m/z 71.06 [58].

#### Flavonoids

Kaempferol 3,7-dihexoside (compound **16**) generated RDA fragments at m/z 313 and 295, indicating that both rings A and B of the flavonoidal structure contained a hexose moiety. It also demonstrated a characteristic ion at m/z 285.23 due to [C_12_H_18_O_10_]^−^ loss. Rhamnocitrin-*O*-hexoside (compound **29**) produced the same fragment ion because of successive loss of methyl and hexose units. These compounds together with kaempferol (compound **46**) exhibited fragment ions at m/z 151.1 and 133.12 due to RDA reaction [85]. Compound **47** revealed a parent peak at m/z 287.24 which is 2 Da higher than that of kaempferol. Therefore, it was characterized as dihydrokaempferol (aromadendrin). It gave RDA fragments at m/z 151.1 and 135.14 [59].

Similarly, rutin (compound **17**) showed a distinguishing ion at m/z 301.23 due to [C_12_H_20_O_9_]^−^ loss. This compound together with quercetin (compound **43**) generated fragment ions at m/z 272.21 and 255.2 owing to eliminating [CHO]^−^ and [CO + H_2_O]^−^, respectively. They also produced RDA fragment at m/z 151.1 [60].

Peak **44** was assigned as taxifolin. It demonstrated its [M − H]^−^ ion at m/z 303.24 and then lost H_2_O and CO from the C ring to generate fragment ions at m/z 285.22 and 275.23, respectively. There were also the ions at m/z 151.1 and m/z 125 which were generated from RDA reaction in the C ring. Additionally, the fragment ion at m/z 175 was generated from [M − H − H_2_O]^−^ at m/z 285.22 due to loss of the B ring moiety. By referring to literature [69], peak **44** was recognized as taxifolin.

Furthermore, apigenin 7-dihexoside (compound **18**), apigenin-7-*O*-hexoside (compound **25**), apigenin 7- hexuronide, ethyl ester (compound **33**) and genkwanin (7-methoxyapigenin) (compound **54**) exhibited a characteristic fragment ion at m/z 269.23 because of elimination of [C_12_H_20_O_10_]^−^ (dihexose), [C_6_H_10_O_5_]^−^ (monohexose), [C_8_H_12_O_6_]^−^ and [CH_2_]^−^ units, respectively. These compounds together with apigenin (compound **49**) generated fragment ions at m/z 151.1 and 117.13 owing to RDA reaction [61].

Meanwhile, isorhamnetin-3-*O*-dihexoside (compound **19**), isorhamnetin-3-*O*-hexuronide (compound **22**) and isorhamnetin-3-*O*-hexoside (compound **24**) exhibited a distinct fragment ion at m/z 315.26 due to elimination of C_12_H_20_O_9_, C_6_H_8_O_6_ and C_6_H_10_O_5_, respectively. All these compounds and isorhamnetin (compound **48**) showed fragment ions at 300.22 and 271.2 due to eliminating [CH_3_]- and [CH_3_ + CHO]^−^, respectively. They also showed the characteristic RDA fragment at m/z 151.1 [62]. Compounds **19**, **22**, and **24** exhibited their sugar moieties in the second RDA fragment that was at m/z 487, for compound **19**; at m/z 339, for compound **22**; and at m/z 325, for compound **24**, indicating that ring A of these compounds were free of sugar moieties.

In addition, naringin (compound **20**), naringenin 7-*O*- hexoside (compound **27**) and naringenin (compounds **51**) were fragmented similarly except that naringin and naringenin 7-*O*- hexoside had extra 308.28 and 162.14 Da corresponding to disaccharide and monosaccharide moieties, respectively. These compounds showed RDA fragments at m/z 151.1 and 119.14, and a fragment at 107.09 corresponding to [151.1-CO_2_]^−^ [63]. Compound **52** having additional 2 Da to the molecular ion of naringenin was suggested to be dihydronaringenin (phloretin). It was affirmed by its RDA fragments at m/z 151.1 and 121.16 [70]. Whereas compound **67** with [M-H]^−^ at m/z 407.48 and fragment ions at m/z 119.14 and 287.33 was proposed to be 6,8-diprenylnaringenin. It was identified by having extra 136 Da in its molecular ion and RDA fragment than those of naringenin [76].

Compounds **23**, **26**, **30**, **32** and **34** were assigned as flavonoidal glycosides. They displayed their flavonoidal aglycones at m/z 285.23, 286.24, 253.23, 283.26 and 267.26, respectively. These compounds were annotated as luteolin-7-*O*-hexoside, cyanidin-3-*O*-deoxyhexoside, daidzein-7-*O*-hexoside, trifolirhizin and ononin, respectively. Compounds **23**, **26**, **30** and **34** were subjected to RDA reaction and generated characteristic fragments at m/z 151.1 and 133.12, for compound **23**; 151.1 and 135.1, for compound **26**; 135.1 and 117.13, for compound **30**; and 132.16 and 135.1, for compound **34** [63]. While compound **32** generated fragments ions at m/z 268.23 and 224.23 owing to loss of methyl and CO_2_ groups, successively [65].

Two isoflavones were identified which are genistein (compound **50**) and glabridin (compound **65**). They displayed their molecular ions at m/z 269.23 and 323.36, successively. Genistein produced fragment ions in MS2 spectrum at m/z 241.22 and 225.23 corresponding to neutral loss of CO and CO_2_, respectively [65]. Whereas glabridin lost [C_7_H_6_O_2_]^−^ to yield fragment ion at m/z 201.24 [75]. On the other hand, compound **53** was assigned to be the methoxylated flavone, tricin. It demonstrated its molecular ion at m/z 329.28 which was subjected to fragmentation to generate ions at m/z 314.25, 299.22 and 271.21 that are related to successive loss of two methyl and carbonyl groups [71].

Moreover, compounds **28**, **31** and **45** displaying molecular ions at m/z 457.36, 441.37 and 305.26 were characterized as epigallocatechin gallate, catechin gallate and epigallocatechin, respectively. The characterization was relied on the fragment ion at m/z 125.1 for the three compounds corresponding to [C_6_H_5_O_3_]^−^, which was originated after two bonds cleavage in ring C and it was composed of the phenolic ring A [64]. Catechin gallate was distinguished from epigallocatechin gallate and epigallocatechin by the existence of the fragment ion [C_6_H_5_O_2_]^−^ at m/z 109.1 which was corresponding to ring B of catechin gallate. Epigallocatechin was differentiated from epigallocatechin gallate and catechin gallate by the lack of the fragment ion [C_7_H_5_O_5_]^−^ at m/z 169.11 indicating the lack of gallate moiety attached to 3-OH [64].

Additionally, chalcone (compound **56**) was recognized by the parent peak at m/z 207.25 and the daughter ion peaks in the MS2 spectrum at m/z 130.14, corresponding to the fragment ion [C_9_H_7_O-H]^−^ which was formed by loss of one phenolic ring, and 102.13, related to cleavage of 1, 2 bond [73]. Compound **55** with additional 32 Da at the molecular ion and fragmented in the same way as chalcone was recognized as 2’,4’-dihydroxychalcone [72].

The most characteristic components of *S. marianum* is silymarin mixture. It was identified in the mass spectra by seven compounds, which are silyamandin (compound **57**), silychristin (compound **59**), silydianin (compound **60**), silybin A (compound **61**), silybin B (compound **62**), isosilybin A (compound **63**) and isosilybin B (compound **64**). Silybin A and B, isosilybin A and B, silydianin and silychristin all had their [M-H]^−^ ions at m/z 481.43, while silyamandin exhibited its [M-H]^−^ ion at m/z 497.43. All seven compounds had similar fragment ions at m/z 463, 453, 179 and 125. Both silybin and isosilybin generated the following fragment ions in common; 435, 301, 283, 273, 257 m/z. However, silybin produced a fragment ion at m/z 423 that was not generated in case of isosilybin. On the other hand, silydianin produced characteristic fragment ions at m/z 409, 151 and 301 m/z. Meanwhile, silychristin produced the following fragment ions at m/z 433, 423, 355, 337 and 325 m/z. Finally, silyamandin exhibited distinguishable peaks at m/z 480, 470, 375 and 355. The fragmentation patterns of these flavonolignans were similar to those explained in literature [74]. Another two compounds were identified which are 2,3-dehydrosilybin (compound **58**) and silandrin (compound **66**). 2,3-dehydrosilybin was suggested by its molecular ion at m/z 479.41 which is 2 Da lower than that of silybin. Whereas silandrin (isosilybin; 3-deoxy) was proposed by its molecular ion at m/z 465.43 which is 16 Da lower than that of silybin.

#### Coumarins

Coumarin (compound **37**) was proposed for the parent ion at m/z 145.14 which was further fragmented to yield ion at m/z 117.13 due to loss of carbonyl group [67]. Compound **36** with extra 16 Da in both parent and daughter ions was identified as 4-hydroxycoumarin [67]. Compounds **39** with additional 14 Da than compound **36** was suggested to be 4-Methylumbelliferone. It fragmented in the same way as compounds **36** and **37** by loss of carbonyl group to give fragment ion at m/z 147.15 [67]. Additionally, compound **42** giving parent peak at m/z 217.2 was proposed to be 4-Methylumbelliferyl acetate. It exhibited daughter ion peak at m/z 175.16 related to elimination of acetyl group [M-H-42.04]^−^ [68]. Up to the authors’ knowledge, it is the first report of coumarins in *S. marianum*.

#### Alcohols

Compound **35** having parent peak at m/z 519.52 was annotated as dehydrodiconiferyl alcohol-hexoside. It was recognized by its daughter ions in the MS/MS spectrum at m/z 357.38 [M-H-162.14]^−^, corresponding to loss of hexoside moiety, 339.36 [M-H-162.14-18.02]^−^, due to lack of H_2_O, 221.23 and 191.2, related to subsequent loss of [C_8_H_8_O_2_]^−^ and methoxy group [66].

Furthermore, compound **38** giving rise to deprotonated ion at m/z 149.17 was annotated as *p-*coumaryl alcohol. Upon fragmentation, it generated a daughter ion peak at m/z 131.15 because of water loss. Similarly, compounds **40** and **41** having molecular ions at m/z 181.21 and fragment ions at m/z 163.19 [M-H-H_2_O]^−^ were identified as 2-hydroxymethyl-5-(2-hydroxypropan-2-yl) phenol and *p*-mentha-1,3,5-triene-2,7,8-triol, respectively [86].

#### Triterpenes

Three triterpenoids were identified. One of them was silymin A (compound **69**) which is ursane- type triterpene exhibiting its parent peak at m/z 483.66 and having daughter peaks at m/z 465.64 and 421.64 due to successive loss of H_2_O and CO_2_ [78]. The other two triterpenoids are 24-methylenelanost-8-ene-3,25,28-triol, 3-*O*-hexoside (compound **68**) and 3,20-dihydroxy-24-methylenelanost-8-en-7-one (compound **70**) which belong to lanostane-type triterpenes. Compound **68** was annotated according to its parent peak at m/z 633.88 and daughter peaks at m/z 471.74, 357.55 and 339.53 related to [M-H-C_6_H_10_O_5_]^-^, [M-H-C_6_H_10_O_5_-C_7_H_14_O]^-^ and [M-H-C_6_H_10_O_5_-C_7_H_14_O-H_2_O]^-^, respectively. Whereas compound **70** demonstrated its parent ion peak at m/z 469.72 and generated daughter peaks at m/z 371.53 and 353.51 related to [M-H-C_7_H_14_]^-^ and [M-H-C_7_H_14_-H_2_O]^-^, successively [77].

#### Fatty acids

12-Tridecene-4,6,8,10-tetraynal (compound **71**) and 1,3-Tridecadiene-5,7,9,11-tetrayne,1,2-epoxide (compound **72**) were recognized by their molecular ions at m/z 179.19. They were differentiated by their different fragmentation patterns demonstrated in their MS2 spectra. Compound **71** showed fragment ions at m/z 151.18 and 137.15 due to successive elimination of carbonyl and methyl groups. Whereas compound **72** produced fragment ions at m/z 164.16 and 149.13, owing to sequential loss of two methyl groups [79].

Moreover, the MS spectra of compounds **73**, **74**, **75**, **76**, **78**, **81**, **82**, **83**, **84** and **85** demonstrated molecular ion peaks at m/z 291.41, 277.42, 279.44, 281.45, 309.51, 227.36, 255.42, 283.47, 311.52 and 339.58, respectively, together with their fragment ions related to neutral loss of H_2_O and CO_2_. These compounds were recognized as 12-oxo-phytodienoic acid, linolenic acid, linoleic acid, oleic acid, gadoleic acid, myristic acid, palmitic acid, stearic acid, arachidic acid and behenic acid, respectively [80].

Furthermore, two fatty acids methyl esters were recognized by their deprotonated ions at m/z 293.46 (compound **79**) and 295.48 (compound **80**). They yield fragment ions in their MS2 spectra at m/z 262.43 (for compound **79**) and 264.45 (for compound **80**) because of methoxy group loss, and at m/z 221.4 corresponding to lack of McLafferty ion which is characteristic to methyl esters [82]. Compound **79** had a base peak at m/z 81.14 due to lack of the hydrocarbon ion [C_6_H_9_]^-^, while compound **80** demonstrated a base peak at m/z 55.1 owing to lack of the hydrocarbon ion [C_4_H_7_]^-^ [83]. Accordingly, these compounds were proposed to be linoleic acid methyl ester (compound **79**) and 16-octadecenoic acid methyl ester (compound **80**).

In addition, compounds **77** and **86** with parent ions at m/z 241.35 and 295.52 were suggested to be 2,9,16-heptadecatriene-4,6-diyn-8-ol and phytol, respectively. These aliphatic alcohols were assured by their fragment ions corresponding to loss of water at m/z 223.33 (for compound **77**) and 277.5 (for compound **86**). Moreover, 2,9,16-heptadecatriene-4,6-diyn-8-ol generated another fragment ion at m/z 226.32 due to lack of terminal methyl group. Whereas phytol exhibited a base peak at m/z 71.14 because of the hydrocarbon ion [C_5_H_11_]^-^ loss [81].

Finally, (R)-gamma-tocotrienol (compound **87**) was identified by its parent ion at m/z 409.63 and fragment ions in the MS/MS spectrum at m/z 394.6 [M-H-CH_3_]^-^, 379.57 [M-H-CH_3_-CH_3_]^-^ and 151.19 [M-H-C_19_H_30_]^-^ [84].

**Fig. 2:**
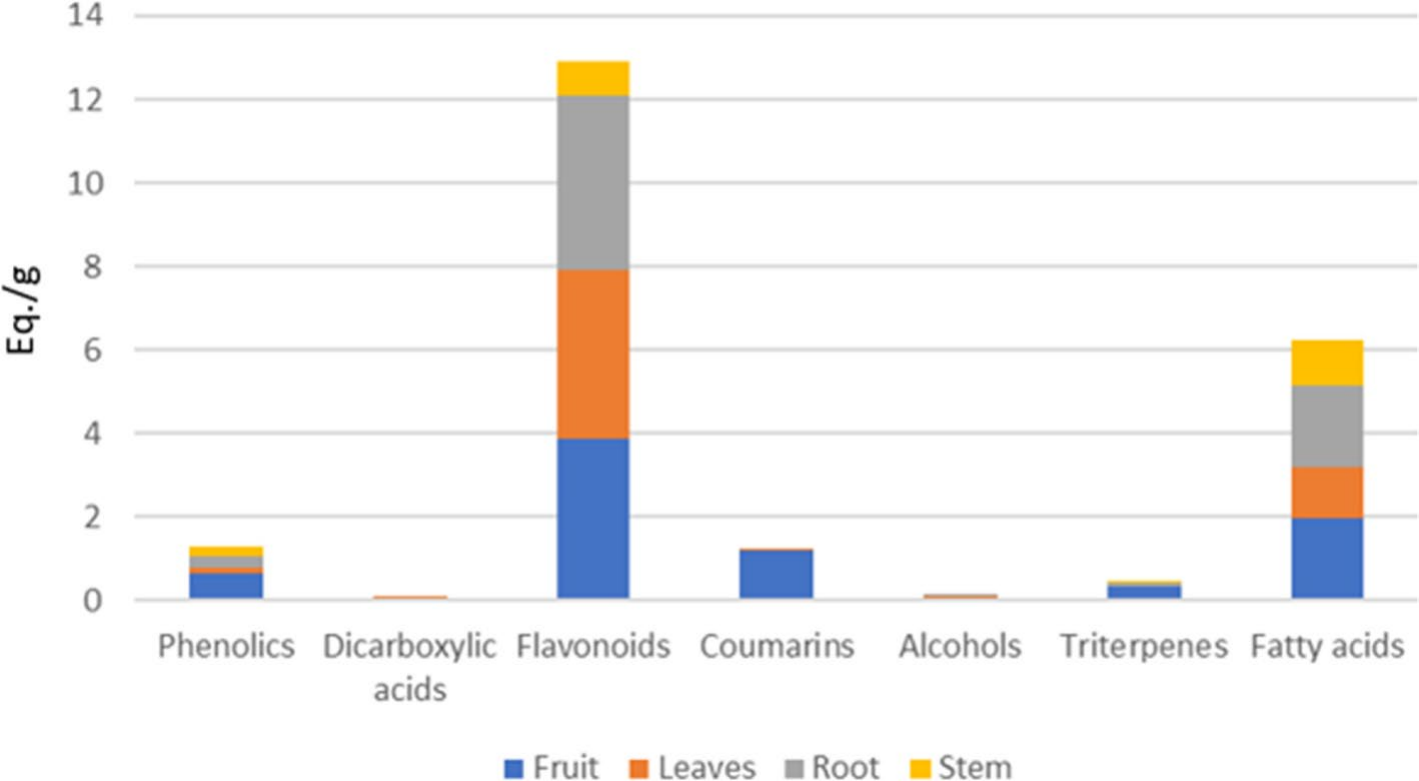
Relative quantitation of the total phenolics, dicarboxylic acids, flavonoids, coumarins, alcohols, triterpenes, and fatty acids in different organs of *S. marianum* expressed as mg Equivalents (Eq.)/g dry weight

Thereafter, all identified compounds in *S. marianum* samples were subjected to relative quantitation via the calibration curves illustrated in Semi-quantitation of identified compounds using UPLC-MS/MS section. and their relative contents are presented in Fig. 2 & Table S1.

### Unsupervised HCA-heat map for chemical profiling of *S. marianum* different organs

**Fig. 3:**
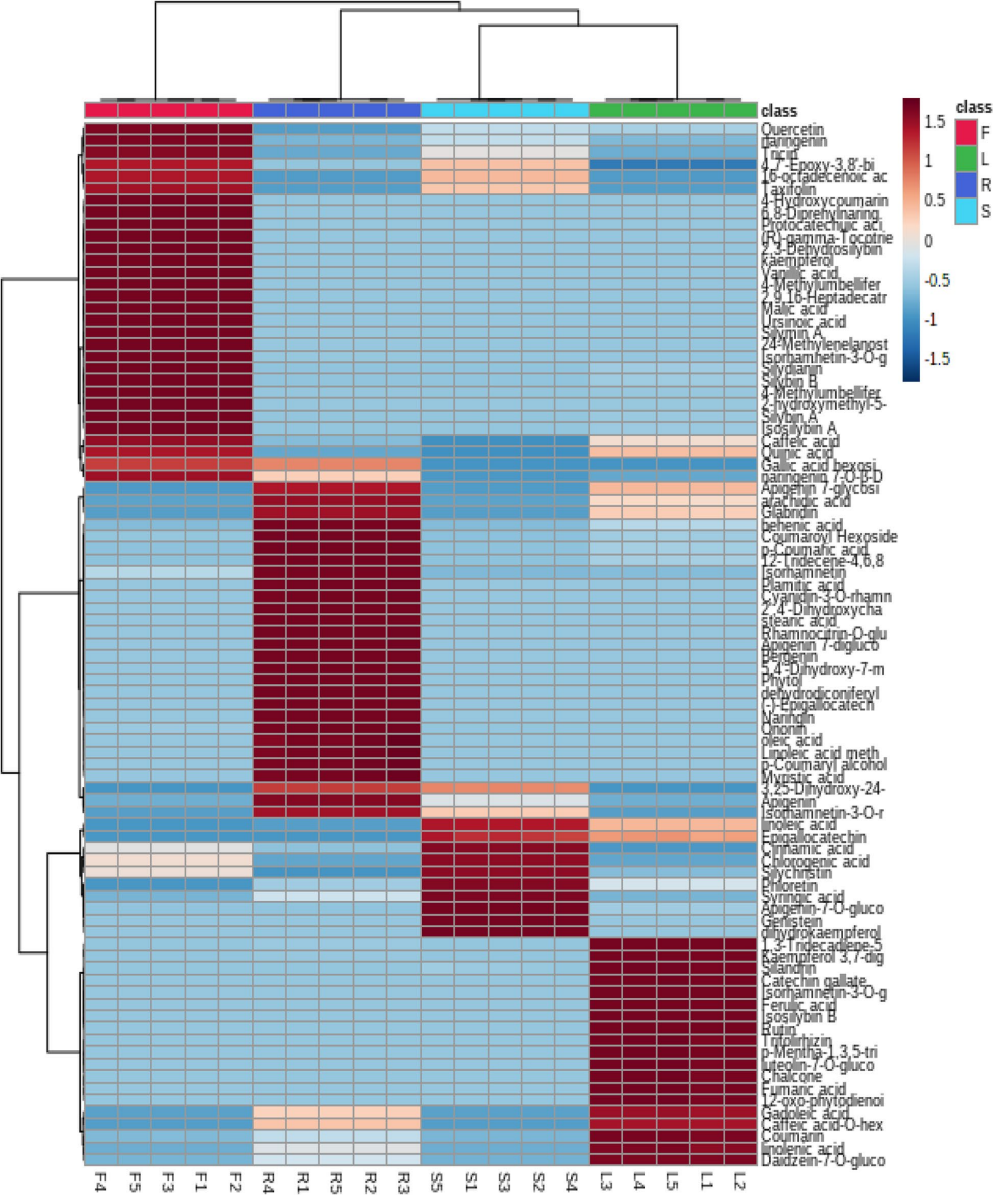
Hierarchical analysis heat maps of all identified metabolites in fruit, leaves, roots and stems of *S. marianum*. Brick red and blue indicate higher and lower abundances, respectively

In this section, comparative chemical profiling of *S. marianum* fruits, leaves, roots and stems was attempted using UPLC-tandem mass analysis combined with multivariable statistical analysis. The semi-quantitative data of characterized compounds in the previous section (Table S1) were implemented to conduct an unsupervised dendritic analysis for the extracts under investigation. As shown in Figs. 1, 2 and 3 considerable variation in chemical profile of milk thistle different organs was observed. Hierarchical clustering analysis (HCA)- heat map (Fig. 3) showed the grouping of the different *S. marianum* organ extracts into three separate clusters, the first was assigned for the five fruit samples, the second allocated for the root samples while the third one was split into two subclusters; namely the leaves and stem samples indicating relative proximity in their chemical composition and proving the previous findings gained by Javeed et al. that calculated the total phenolic and flavonoid contents in different milk thistle parts and revealed that the leaves and stems extracts were enriched with higher amounts of them [87].

It was observed that *S. marianum* fruit samples possessed the highest relative content of flavonolignans such as 2,3-dehydrosilybin, silymin A, silydianin, isosilybin A, silybin A and B, and this finding is in a good agreement with that reported by Korany et al. [39]. Other metabolic classes were diversely distributed among different organ clusters and subclusters as indicated by the dark red color code (Fig. 3). Four compounds were found in all the studied milk thistle organs, namely the phenolic acid “cinnamic acid” which recorded highest accumulation in stems and two flavonoidal aglycones namely naringenin and tricin which were both highly accumulated in fruits followed by seeds, and finally the fatty acid ester “16-octadecenoic acid methyl ester” which was also detected in greater amount in milk thistle fruits. Meanwhile, close observation of Fig. 3. revealed that the two coumarins 4-methylumbelliferone and 4-hydroxycoumarin in addition to 6,8-diprenylnaringenin, 2-hydroxymethyl-5-(2-hydroxypropan-2-yl) phenol and the two flavonolignans silybin A and B were the main constituents in fruit samples. In contrast, milk thistle leaves samples exhibited greater accumulation of the phenolic acid glycoside “caffeic acid-*O*-hexoside” and the aglycone “quinic acid”. Further, isorhamnetin, gallic acid hexoside, bergenin, caffeic acid-*O*-hexoside, and apigenin were the major compounds found in roots samples. Finally, cinnamic and syringic acids, genistein, apigenin-7-*O*-hexoside, taxifolin and phloretin were the main detected constituents in milk thistle stems.

It is worthy to mention that, up to the authors’ knowledge, this is the foremost comprehensive evaluation of *S. marianum* different organs chemical profiles.

### OPLS-DA for supervised multivariate discrimination between different organs

**Fig. 4:**
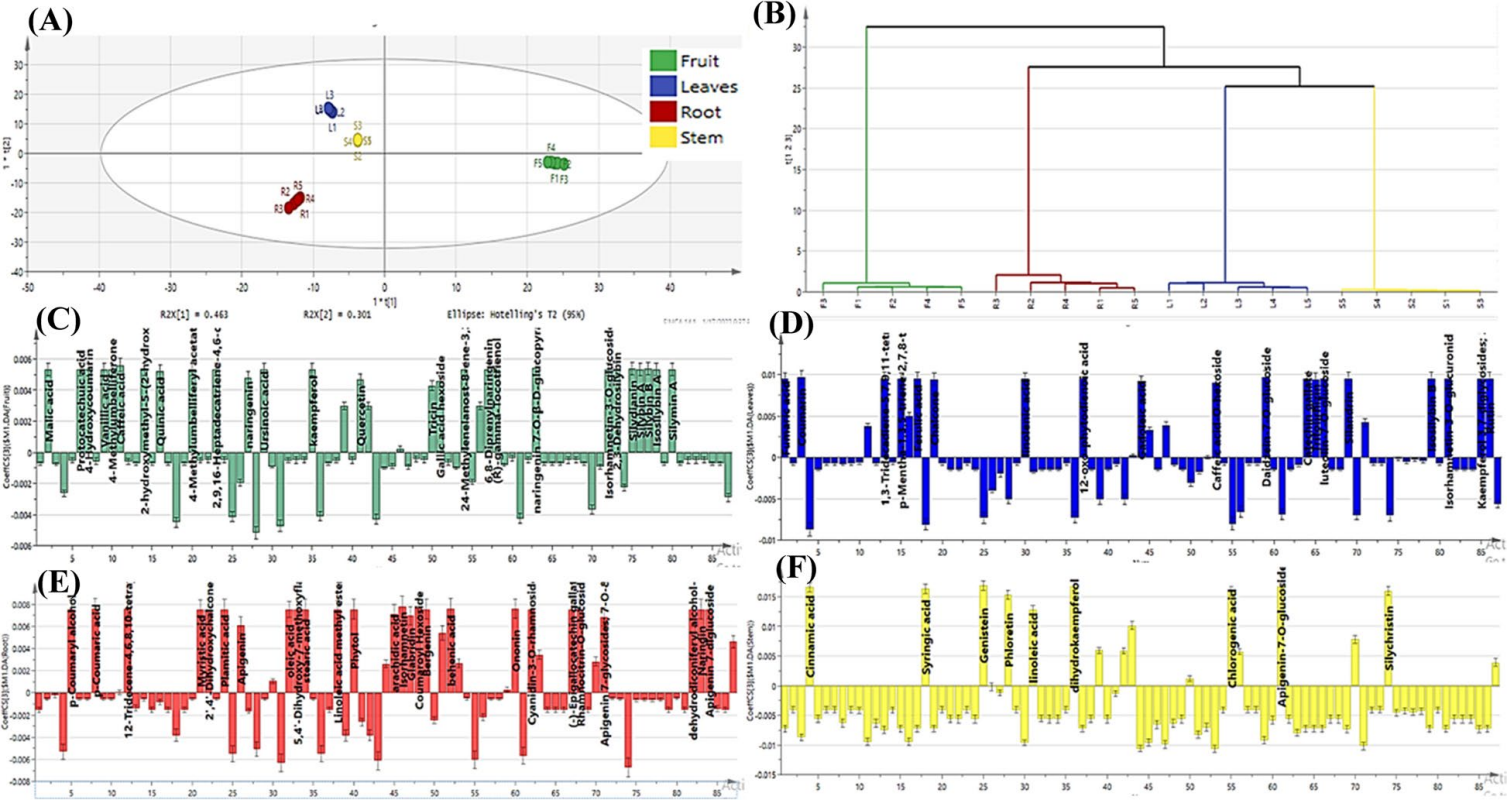
OPLS-DA scatter plot (t1 scores vs. t2 scores) (**A**), Dendrogram derived from the hierarchical cluster analysis, based on the Ward method of fruits, leaves, roots and stems samples of *S. marianum* (**B**), Coefficient plots of the OPLS-DA model of *S. marianum* fruits (**C**), leaves (**D**), roots (**E**), and stems (**F**) to determine discriminative metabolites

For the sake of inter- and intra-class discrimination of fruits, leaves, roots and stems samples; an OPLS-DA multivariate model was created utilizing the MS data obtained from LC-MS/MS analysis (Fig. 4A and B). Moreover, OPLS-DA was able to unravel the discriminatory markers characteristic for each class chemical profile via coefficient plots of each organ separately (Fig. 4C and F). The first and second latent variables of the constructed model accounted for 46.3% and 30.1% of the variability, respectively. Moreover, the model exhibited high reliability and prediction ability represented by high goodness of fitness (R^2^ = 0.998) and goodness of prediction (Q^2^ = 0.996). For validation of the current OPLS-DA model; permutation plots for fruits, leaves, roots and stems (Fig. S1) using 20 permutations for each class were constructed. The blue regression line of Q^2^ points intersected with vertical axis below the zero, while the green R^2^ values to the left were lower to the original point to the right which strongly indicated the model validity. ROC curves (Fig. S2) were constructed, and AUC were found to be equal to one for all classes indicating the excellent classification power of the model.

In between class discrimination along the first latent variable (LV_1_) was observed in the 2D score scatter plot (Fig. 4A) where all the fruit samples where successfully grouped along its positive side, while other organ samples were on the negative side of LV_1_. Whereas the second latent variable (LV_2_) successfully separated the root samples on its negative side from the leaves and stem samples on the positive side of the same LV. This classification was in agreement with that observed OPLS-DA dendrogram (Fig. 4B) where it revealed the existence of two principal clusters; one for the fruit samples and the other was additionally sub-clustered into a subcluster for roots and another one comprised of tested stems and leaves samples. The OPLS-DA coefficients plots (Fig. 4C and F) allowed the recognition of phytoconstituents responsible for the segregation of each milk thistle organ samples into separate class. Caffeic acid, naringenin 7-*O*-hexoside, silydianin, silybin B, isosilybin A and silybin A were the main differentiating markers certainly correlated to fruit samples (Fig. 4C). Meanwhile, daidzein-7-*O*-hexoside, silandrin, linolenic acid, 1,3-tridecadiene-5,7,9,11-tetrayne 1,2-epoxide, kaempferol 3,7-dihexoside, isorhamnetin-3-*O*-hexuronide and isosilybin B were found to be the foremost constituents related to leaves samples class (Fig. 4D). In contrast, the flavonoidal aglycone isorhamnetin, coumaroyl hexoside, behenic acid, 12-tridecene-4,6,8,10-tetraynal, *p*-coumaric acid and ononin were the positively related compounds to root class (Fig. 4E). Moreover, the flavones genistein, dihydrokaempferol and apigenin-7-*O*-hexoside, the phenolic acids cinnamic, syringic and chlorogenic acid, silychristin, phloretin and linoleic acid were the principal differentiating markers showing positive correlation to stems class (Fig. 4F).

### Selective virucidal activity of the tested *S. marianum* organs extracts against human coronavirus (HCoV-229E) using cytopathic effect (CPE) inhibition assay

**Fig. 5:**
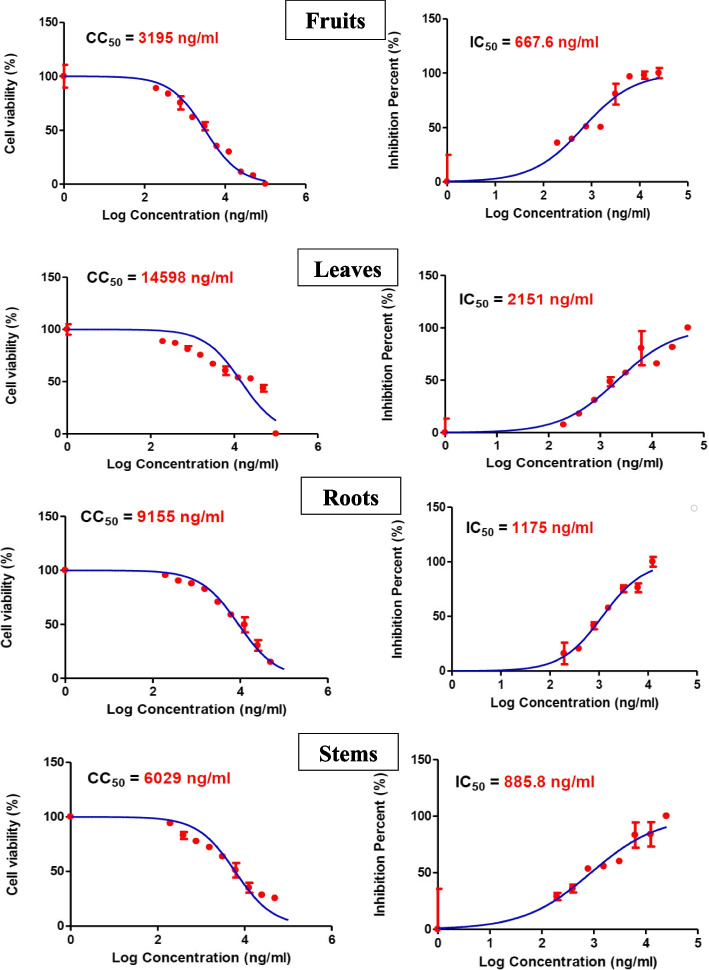
50% cytotoxic concentration (CC50) on Vero E6 cells on the left, and 50% inhibitory concentration (IC50) on HCoV-229E virus on the right of different *S. marianum *organs extracts

The appearance of drug-resistant respiratory viral strains to currently used antivirals such as oseltamivir, zanamivir, peramivir, and laninamivir [88] makes the development of natural selective alternatives with diminished toxicity urgently required. In this context, selective virucidal activity of the tested milk thistle organs extracts against human coronavirus (HCoV-229E) using cytopathic effect (CPE) inhibition assay was performed for the first time. The CPE-inhibition assay was used to identify potential antivirals against human coronavirus 229E. The dose-response assay was designed to determine the range of efficacy for the chosen antiviral, i.e. the 50% inhibitory concentration (IC50), as well as the range of cytotoxicity (CC50). This assay is a critical and a well-reputable tool to assess the efficacy of several synthetic and natural agents against many viruses such as metapneumoviruses [89], influenza viruses [90], enteroviruses [91], and herpes simplex virus [92], among others. Selectivity index (SI = cytotoxicity/bioactivity) appeared to be an indispensable parameter to evaluate during the exploring process of novel antiviral candidates rather than focusing only on pharmacological or toxicological parameters separately [93]. As revealed in Fig. 5 all the tested milk thistle organs samples exhibited dose dependent inhibitory activity on HCoV-229E in nanomolar range. The results were compared to the positive control (remdesivir®) (Table 3). Comparison of the IC50 values of organs samples disclosed that fruit samples had the smallest IC50 value among all tested organs of 667.6 ± 0.5 ng/mL indicating its higher activity against the tested HCoV-229E virus while the leaves possessed the largest IC50 value of 2151 ± 0.9 ng/mL. On the contrary, low 50% cytotoxic concentration (CC50) on Vero E6 cells represent an indication of high toxicity of the tested samples on normal cells. Milk thistle fruits possessed the lowest CC50 of 3195 ± 0.3 ng/mL indicating the highest cytotoxicity among other samples (Fig. 5). Meanwhile, the leaves recorded the lowest toxicity with CC50 of 14,598 ± 1.2 ng/mL.

**Table 3:** Cytotoxicity (pCC50, CC50 in g/L) on Vero E6 cells, HCoV-229E antiviral activity (pIC50, IC50 in g/L), and selectivity indices (SI = pCC50/pIC50) of different *S. marianum* organs samples

Organ samples	pCC50	pIC50	SI
Fruits	2.50	3.18	0.79
Leaves	1.84	2.67	0.69
Roots	2.03	2.93	0.70
Stems	2.22	3.05	0.73
Positive control (remdesivir®)	2.55	2.61	0.98

Selectivity index (SI) was then calculated by dividing cytotoxicity as pCC50 (-log CC50 in g/L) on HCoV-229E antiviral activity as pIC50 (-log IC50 in g/L) to inspect the samples of high selectivity to virus infected cells without causing toxicity to normal cells (Table 3). The lower the selectivity index the more selective the tested sample. Leaves samples possessing low pCC50 and high pIC50 values, that subsequently yielded low selectivity index, are promising anti- human coronavirus 229E drug-like candidates. Although many other researchers have documented the antiviral efficacy of silymarin and milk thistle supplements [33, 94–96], the present study is the first to compare the antiviral efficacy of different milk thistle organs aiming at valorizing the unused plant parts.

### Correlation analysis to selective antiviral activity for unraveling bioactivephytoconstituents from the tested *S. marianum* organs samples

**Fig. 6:**
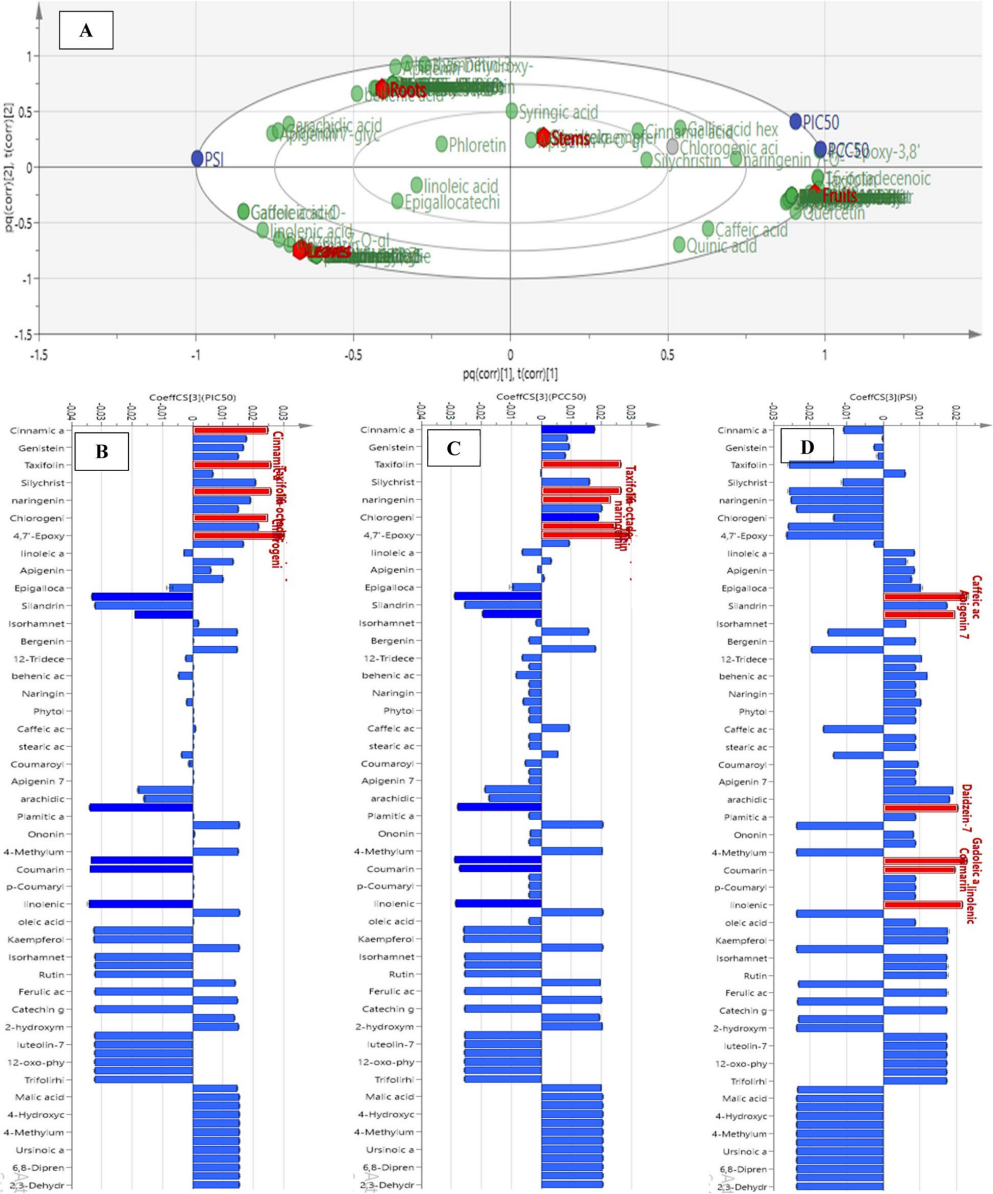
Orthogonal Projections to Latent Structures (OPLS) biplot of the tested samples in correlation to the bioactive markers (**A**). Coefficient plots of OPLS model in order to determine biomarkers responsible for the antiviral activity (PIC50) (**B**), cytotoxicity (PCC50) (**C**), and selectivity (PIS) (**D**)

OPLS model and its accompanying correlation coefficient analysis were implemented for detection of significant phytoconstituents having selective virucidal activity against human coronavirus (HCoV-229E) amongst the four milk thistle organ samples studied, as well as evaluating consequent classification of the samples based on bioactivity. The biplot of the constructed OPLS model (Fig. 6A) exposed in-between class discrimination of fruits and stems from roots and leaves samples where the first exhibited spatial relation to cytotoxicity represented as pCC50 and antiviral activity on HCoV-229E as pIC50 while the later classes were in proximity to PSI indicating better selectivity. Further, studying the coefficient plots (Fig. 6B and D) portrayed that 16-octadecenoic acid methyl ester, taxifolin, cinnamic and chlorogenic acids were shown to be the constituents possessing the highest positive correlation to HCoV-229E inhibitory activity (Fig. 6B). While 16-octadecenoic acid methyl ester, taxifolin, tricin and naringenin were the major metabolites positively related to cytotoxic activity on normal cells (Fig. 6C). Finally, Fig. 6D indicated that caffeic acid-*O*-hexoside, gadoleic and linolenic acids, daidzein-7-*O*-hexoside, apigenin-7-*O*-hexoside ethyl ester and coumarin were the most potentially selective anti-human coronavirus 229E phytoconstituents.

These findings are consistent with previous research that found that octadecenoic acid derivatives can bind to several coronaviruses’ proteins such as RNA-dependent RNA polymerase, main protease, and spike protein S1 to degrees similar to those possessed by the known antiviral drug umifenovir [97]. It also exhibited activity against influenza A and B viruses [98]. In addition, taxifolin showed the ability to inhibit the replication of HCoV-229E in Huh-7 cells at 2.5 µM and this inhibitory activity augmented with increasing its concentration. This activity was explained by its ability to inhibit the viral main protease activity [99]. Moreover, chlorogenic, caffeic, linolenic acids, and daidzein were found to inhibit HCoV S-glycoprotein attachment to host cells. This was illustrated by their ability to impair the function of HSPA5 SDBβ, which is the binding site for viral S-glycoprotein [100]. Furthermore, tricin was found to have antiviral activities against influenza A and B strains by inhibiting viral mRNA synthesis [101]. Besides, naringenin was found to inhibit cytopathic effect in Vero E6 cells infected with SARS-CoV-2 in a time and concentration-dependent manner. This effect was explained by its ability to inhibit endo-lysosomal Two-Pore Channels (TPCs), a pathway facilitating viral entry to host cell [101]. Further, apigenin and coumarins were found to be SARS-CoV-2 main protease inhibitors, thus inhibiting viral replication in the host cell [102, 103].

## Conclusion

This study provides the first comparative evaluation of the metabolomes of *S. marianum* different organs applying UPLC-MS/MS coupled with multivariate analysis. HCA-heat map and OPLS-DA revealed in-between class discrimination between fruits and the other organs samples, in addition to within class discrimination between root samples which were separated from the leaves and stem samples. The OPLS-DA coefficients plots allowed the recognition of phytoconstituents responsible for the segregation of each organ samples into separate class. All studied *S. marianum* organs extracts were tested for selective virucidal activity against human coronavirus (HCoV-229E), and they all exhibited dose dependent inhibitory activity in nanomolar range with variable degrees of safety, efficacy, and selectivity. OPLS model and its accompanying correlation coefficient analysis were implemented for detection of significant phytoconstituents having effective, safe, and selective antiviral potential amongst the four studied *S. marianum* organs. The study in hand valorizes the importance of different *S. marianum* organs as wealthy sources of valuable antiviral agents. The future work will be the isolation of the recognized promising antiviral phytoconstituents from different milk thistle organs, followed by extensive in vitro and in vivo testing of their biological activities to afford more conclusive and comprehensive therapeutic approaches that enable to introduce these drugs to the market.

### Supplementary Information

**Supplementary Material 1.**

## Data Availability

The datasets generated and analyzed during the current study are all mentioned in the presented manuscript.

## Abbreviations

ESI: Electrospray ionization
TQD: Tandem Quadropole Detection
HCoV-229E: Human coronavirus 229E
OPLS-DA: Orthogonal Projections to Latent Structures-Discriminant Analysis
OPLS: Orthogonal Projections to Latent Structures
HCA: Hierarchical Clustering Analysis
CPE: Cytopathic Effect
COVID-19: Coronavirus Disease 2019
SARS-CoV-2: Severe Acute Respiratory Syndrome Coronavirus 2
STAT3: Signal Transducer and Activator of Transcription 3
RdRp: RNA-dependent RNA polymerase
HCoV: Human Coronavirus
MERS-CoV: Middle East Respiratory Syndrome Coronavirus
Vero E6 cells: African green monkey kidney
CID: Collision-Induced Dissociation
ANOVA: One-Way Analysis of the Variance
DMEM: Dulbecco's Modified Eagle Medium
FBS: Fetal Bovine Serum
CCID 50: 50 percent cell culture infective dose
PBS: Phosphate-Buffered Saline
OD: Optical Density
CC50: 50% Cytotoxic Concentration
IC50: 50% Inhibitory Concentration
RDA reaction: Retro-Diels–Alder reaction
R^2^: Goodness of fitness
Q2: Goodness of prediction
ROC curve: Receiver Operating Characteristic curve
AUC: Area under the curve
LV: Latent Variable
SI: Selectivity Index
HSPA5: Heat shock protein family A member 5
TPCs: Two-pore channels
