# Supplementary material for: Metabolic profiling of milk thistle different organs using UPLC-TQD-MS/MS coupled to multivariate analysis in relation to their selective antiviral potential
Source: BMC Complement Med Ther. 2024 Mar 7;24:115. doi: 10.1186/s12906-024-04411-7 (PMC10921647; doi:10.1186/s12906-024-04411-7)
Supplement: Supplementary file 1 — Supplementary Material 1. [file 12906_2024_4411_MOESM1_ESM.docx]

**Supplementary material**

**Table S1: Content of identified compounds in different organs of *S. marianum* analyzed by UPLC-MS/MS (data are expressed as mg mL^-1^)**

|  | **Metabolites** | **Fruit** | **Leave** | **Root** | **Stem** |
| --- | --- | --- | --- | --- | --- |
| **1** | Gallic acid hexoside | 0.177437977 | 0 | 0.147889049 | 0 |
| **2** | Bergenin | 0 | 0 | 0.054038177 | 0 |
| **3** | Caffeic acid-*O*-hexoside | 0 | 0.095031657 | 0.04691638 | 0 |
| **4** | Quinic acid | 0.071819537 | 0.038245489 | ˂ LOD | 0 |
| **5** | Protocatechuic acid | 0.196724697 | 0 | 0 | 0 |
| **6** | Vanillic acid | 0.076165734 | 0 | 0 | 0 |
| **7** | Syringic acid | 0 | 0 | 0.012980517 | 0.079394806 |
| **8** | Caffeic acid^*^ | 0.055077426 | 0.019930214 | ˂ LOD | 0 |
| **9** | p-Coumaric acid | 0 | 0 | 0.006676229 | 0 |
| **10** | Ferulic acid | 0 | 0.007154003 | 0 | 0 |
| **11** | Cinnamic acid | 0.034066166 | ˂ LOD | 0.00896828 | 0.108437263 |
| **12** | Chlorogenic acid | ˂ LOD | 0 | 0 | 0.018672051 |
| **13** | Ursinoic acid | 0.023879083 | 0 | 0 | 0 |
| **14** | Malic acid^*^ | 0.032623912 | 0 | 0 | 0 |
| **15** | Fumaric acid | 0 | 0.034145518 | 0 | 0 |
| **16** | Kaempferol 3,7-dihexoside | 0 | 0.14490743 | 0 | 0 |
| **17** | Rutin | 0 | 0.116188869 | 0 | 0 |
| **18** | Apigenin 7-dihexoside | 0 | 0 | 0.171367664 | 0 |
| **19** | Isorhamnetin-3-*O*-dihexoside | 0 | 0 | 0.10689972 | 0.144031888 |
| **20** | Naringin | 0 | 0 | 0.135460393 | 0 |
| **21** | Coumaroyl hexoside | 0 | 0 | ˂ LOD | 0 |
| **22** | Isorhamnetin-3-*O*-hexuronide | 0 | 0.078889047 | 0 | 0 |
| **23** | Luteolin-7-*O*-hexoside | 0 | 0.17217215 | 0 | 0 |
| **24** | Isorhamnetin-3-*O*-hexoside | ˂ LOD | 0 | 0.180514907 | 0 |
| **25** | Apigenin-7-*O*-hexoside | 0 | 0.180409897 | 0 | ˂ LOD |
| **26** | Cyanidin-3-*O*-deoxyhexoside | 0 | 0 | 0.181396411 | 0 |
| **27** | Naringenin 7-*O*- hexoside | ˂ LOD | 0.17864714 | 0.082025916 | 0 |
| **28** | (-)-Epigallocatechin gallate | 0 | 0 | 0.131222991 | 0 |
| **29** | Rhamnocitrin-*O*-hexoside | 0 | 0 | 0.170121953 | 0 |
| **30** | Daidzein-7-*O*-hexoside | 0 | 0.131930944 | 0.177549019 | 0 |
| **31** | Catechin gallate | 0 | 0.157713617 | 0 | 0 |
| **32** | Trifolirhizin | 0 | 0.179529327 | 0 | 0 |
| **33** | Apigenin 7- hexuronide, Et ester | 0 | 0.161807178 | 0.140167757 | 0 |
| **34** | Ononin | 0 | 0 | 0.180657374 | 0 |
| **35** | Dehydrodiconiferyl alcohol-hexoside | 0 | 0 | 0.032217587 | 0 |
| **36** | 4-Hydroxycoumarin | 0.564414937 | 0 | 0 | 0 |
| **37** | Coumarin^*^ | 0 | 0.003867789 | ˂ LOD | 0 |
| **38** | p-Coumaryl alcohol^*^ | 0 | 0 | 0.039671112 | 0 |
| **39** | 4-Methylumbelliferone | 0.601272027 | 0.006894955 | ˂ LOD | 0 |
| **40** | 2-Hydroxymethyl-5-(2-hydroxypropan-2-yl)phenol | ˂ LOD | 0.037715428 | 0 | 0 |
| **41** | p-Mentha-1,3,5-triene-2,7,8-triol | 0 | 0.03785747 | 0 | 0 |
| **42** | 4-Methylumbelliferyl acetate | 0.039506133 | 0 | 0 | 0 |
| **43** | Quercetin^*^ | ˂ LOD | 0.117819364 | 0 | 0.097817794 |
| **44** | Taxifolin | 0.055768505 | 0 | 0 | 0.048173495 |
| **45** | Epigallocatechin | 0 | 0.186636215 | 0 | 0 |
| **46** | Kaempferol | ˂ LOD | 0 | 0 | 0 |
| **47** | Dihydrokaempferol (Aromadendrin) | 0 | 0 | 0 | 0.133882215 |
| **48** | Isorhamnetin | 0.106727019 | 0.183114009 | ˂ LOD | 0 |
| **49** | Apigenin | 0 | 0 | 0.045088178 | 0.143801888 |
| **50** | Genistein | 0 | 0 | 0 | ˂ LOD |
| **51** | Naringenin | ˂ LOD | 0.141447794 | 0.145227056 | 0.08829014 |
| **52** | Phloretin | 0 | 0.15377343 | 0.167582822 | 0.078610178 |
| **53** | Tricin | 0.014027439 | 0.140077757 | 0.141519215 | ˂ LOD |
| **54** | 5,4'-Dihydroxy-7-methoxyflavone (Genkwanin, 7-methoxyapigenin) | 0 | 0 | 0.152017953 | 0 |
| **55** | 2',4'-Dihydroxychalcone | 0 | 0 | 0.164325523 | 0 |
| **56** | Chalcone | 0 | 0.177327037 | 0 | 0 |
| **57** | Silyamandin | 0.145354523 | 0.174675748 | 0.157797785 | 0 |
| **58** | 2,3-Dehydrosilybin | 0.243334121 | 0 | 0 | 0 |
| **59** | Silychristin | 0.145354523 | 0.175219981 | 0 | 0.081230262 |
| **60** | Silydianin | 0.243334121 | 0.167281981 | 0 | 0 |
| **61** | Silybin A | 1.633400682 | 0.153443991 | 0 | 0 |
| **62** | Silybin B | 1.044936411 | 0.133359561 | 0 | 0 |
| **63** | Isosilybin A | 0.072172159 | 0.184156318 | 0 | 0 |
| **64** | Isosilybin B | 0 | 0.091699421 | 0 | 0 |
| **65** | Glabridin | 0 | 0.181361159 | 0.172542738 | 0 |
| **66** | Silandrin | 0 | 0.172112028 | 0 | 0 |
| **67** | 6,8-Diprenylnaringenin | 0.167019411 | 0 | 0 | 0 |
| **68** | 24-Methylenelanost-8-ene-3,25,28-triol, 3-*O*-hexoside | 0.101252668 | 0 | 0 | 0 |
| **69** | Silymin A | 0.223729472 | 0 | ˂ LOD | 0 |
| **70** | 3,20-Dihydroxy-24-methylenelanost-8-en-7-one | 0 | 0 | 0.074806319 | 0.062298504 |
| **71** | 12-Tridecene-4,6,8,10-tetraynal | 0 | 0.04552455 | 0.603717145 | 0 |
| **72** | 1,3-Tridecadiene-5,7,9,11-tetrayne, 1,2-epoxide | 0 | 0.565957105 | 0.012143896 | 0 |
| **73** | 12-oxo-phytodienoic acid | 0 | 0.114815344 | 0 | 0 |
| **74** | Linolenic acid | 0 | 0.051854903 | 0.017486057 | 0 |
| **75** | Linoleic acid^*^ | 0 | 0.210118012 | 0.013210474 | 0.351570113 |
| **76** | Oleic acid | 0 | 0 | 0.013420881 | 0 |
| **77** | 2,9,16-Heptadecatriene-4,6-diyn-8-ol | 0.412551968 | 0 | 0 | 0 |
| **78** | Gadoleic acid | 0 | 0.11129046 | 0.05980507 | 0 |
| **79** | Linoleic acid methyl ester | 0 | 0 | 0.023559773 | 0 |
| **80** | 16-octadecenoic acid methyl ester | 1.30061541 | 0.024758572 | 0.028811141 | 0.771760841 |
| **81** | Myristic acid | 0 | 0 | 0.034438025 | 0 |
| **82** | Plamitic acid | 0 | 0 | 0.089249166 | 0 |
| **83** | Stearic acid | 0 | 0 | 0.191635023 | 0 |
| **84** | Arachidic acid | 0 | 0.048911341 | 0.10362048 | 0 |
| **85** | Behenic acid | 0 | 0.064836224 | 0.435643763 | 0 |
| **86** | Phytol | 0 | 0 | 0.291768579 | 0 |
| **87** | (R)-gamma-Tocotrienol | 0.255056237 | 0 | 0 | 0 |

^*The standards used for retention times comparison and semi-quantitation of the annotated compounds were caffeic acid, malic acid, quercetin, coumarin, p-coumaryl alcohol, lanosterol, and linoleic acid.^


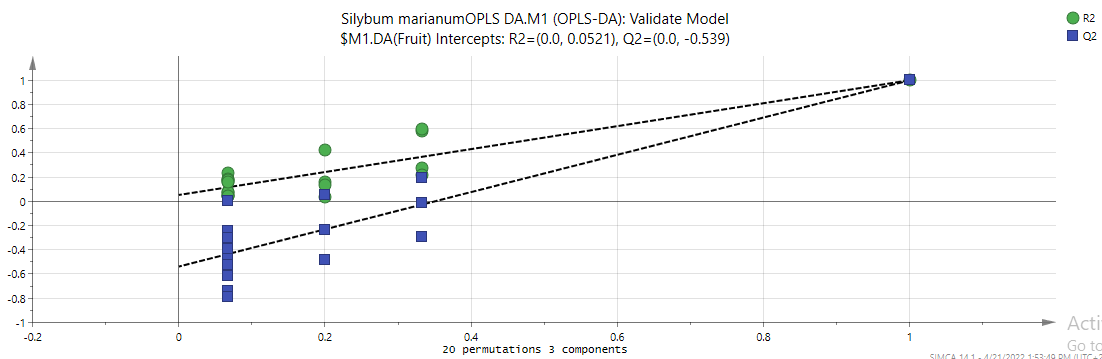

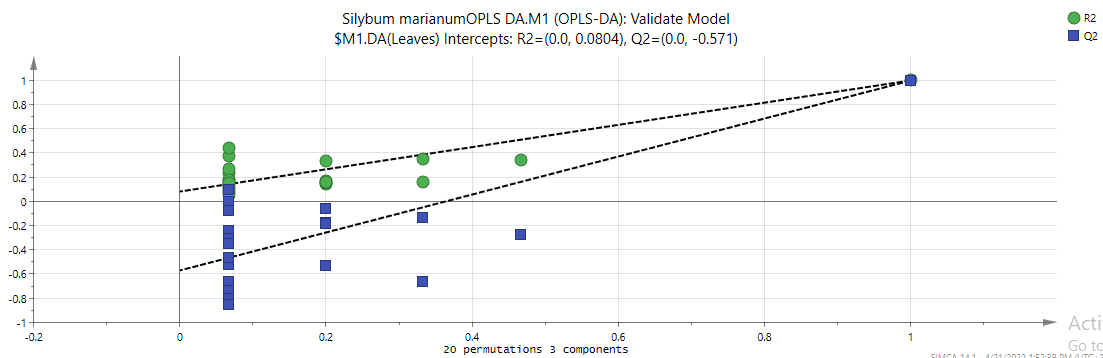

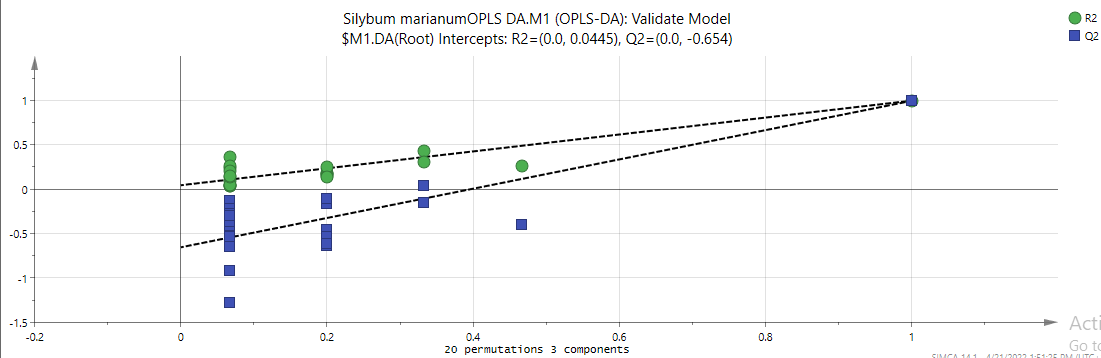


Fruits

Leaves

Roots


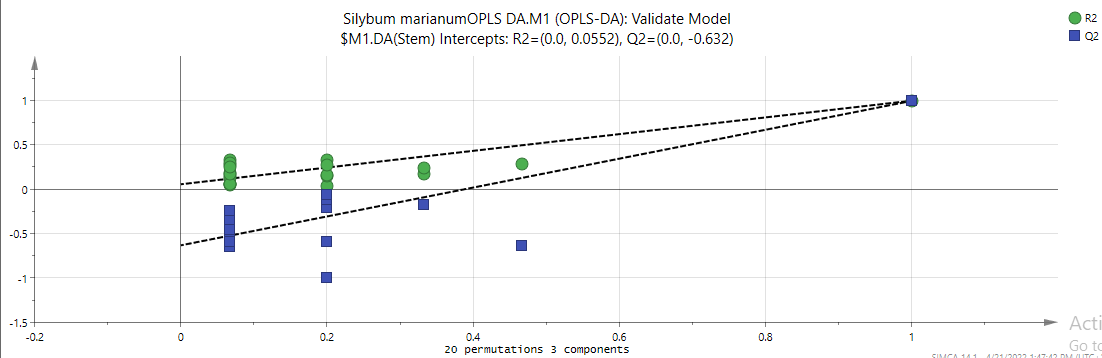


Stems

**Fig. S1.** Permutation plots of supervised OPLS-DA model.


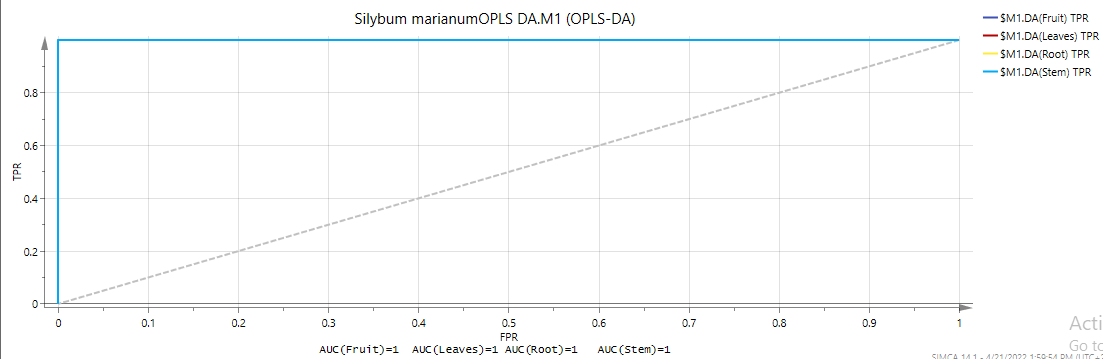


| **Fig. S2.** ROC curves of OPLS-DA |  |
| --- | --- |
